# Roles of APOBEC3A and APOBEC3B in Human Papillomavirus Infection and Disease Progression

**DOI:** 10.3390/v9080233

**Published:** 2017-08-21

**Authors:** Cody J. Warren, Joseph A. Westrich, Koenraad Van Doorslaer, Dohun Pyeon

**Affiliations:** 1 Department of Immunology and Microbiology, University of Colorado School of Medicine, Aurora, CO 80045, USA; cody.warren@colorado.edu (C.J.W.); joseph.westrich@ucdenver.edu (J.A.W.); 2 BIO5 Institute, School of Animal and Comparative Biomedical Sciences, University of Arizona, Tucson, AZ 85721, USA; vandoorslaer@email.arizona.edu; 3 Department of Medicine, University of Colorado School of Medicine, Aurora, CO 80045, USA

**Keywords:** papillomavirus, human papillomavirus (HPV), apolipoprotein B messenger RNA-editing, enzyme-catalytic, polypeptide-like 3 (APOBEC3), innate immunity, virus restriction, cancer mutagenesis, cancer progression, somatic mutation, virus evolution

## Abstract

The apolipoprotein B messenger RNA-editing, enzyme-catalytic, polypeptide-like 3 (APOBEC3) family of cytidine deaminases plays an important role in the innate immune response to viral infections by editing viral genomes. However, the cytidine deaminase activity of APOBEC3 enzymes also induces somatic mutations in host genomes, which may drive cancer progression. Recent studies of human papillomavirus (HPV) infection and disease outcome highlight this duality. HPV infection is potently inhibited by one family member, APOBEC3A. Expression of APOBEC3A and APOBEC3B is highly elevated by the HPV oncoproteins E6 and E7 during persistent virus infection and disease progression. Furthermore, there is a high prevalence of APOBEC3A and APOBEC3B mutation signatures in HPV-associated cancers. These findings suggest that induction of an APOBEC3-mediated antiviral response during HPV infection may inadvertently contribute to cancer mutagenesis and virus evolution. Here, we discuss current understanding of APOBEC3A and APOBEC3B biology in HPV restriction, evolution, and associated cancer mutagenesis.

## 1. Introduction

**Figure 1:**
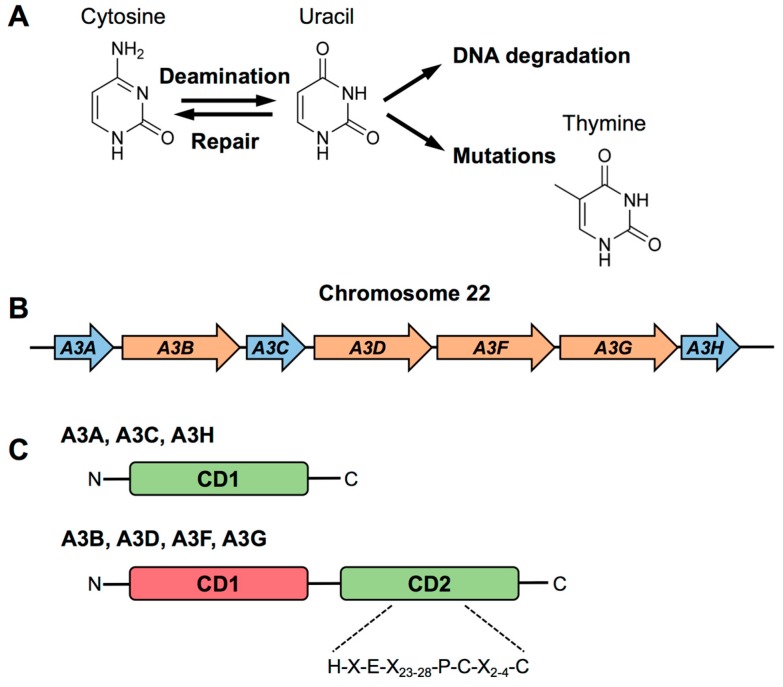
Structural features of the human apolipoprotein B messenger RNA-editing, enzyme-catalytic, polypeptide-like 3 (APOBEC3s). (**A**) APOBEC3 family members convert cytosine to uracil and induce DNA degradation or mutations if the APOBEC3-mediated conversion of cytosine to uracil is not repaired; (**B**) All *APOBEC3* family members are arrayed in tandem on chromosome 22; (**C**) Schematic of APOBEC3 family members containing one or two cytidine deaminase (CD) domains. The catalytically active (green) and inactive (red) cytidine deaminase domains are pictured. The conserved zinc-coordinating motif is pictured between dashed lines.

The family members of apolipoprotein B messenger RNA-editing, enzyme-catalytic, polypeptide-like 3 (APOBEC3; A3) are DNA cytidine deaminases that remove the amino group from a cytosine, converting it to uracil. Cytosine deamination by A3 results in DNA degradation or mutations if not repaired (Figure 1A) [1,2,3]. For many years following their initial discovery, the A3 family members APOBEC3A (A3A) and APOBEC3B (A3B) were considered as viral restriction factors, important only for inhibiting the replication of endogenous retroviruses and retroelements [4,5,6,7]. However, several studies from our and other groups have revealed a broader range of viruses restricted by A3A and A3B: human immunodeficiency virus 1 (HIV-1) [8,9,10], parvovirus [11,12,13], herpesvirus [14,15], hepatitis B virus (HBV) [16,17], and human papillomavirus (HPV) [18,19,20] (Table 1). In addition, recent studies have identified additional important roles for these family members in diverse cellular processes, including (1) promoting catabolism of foreign DNA [21,22]; (2) editing of mRNA transcripts [23,24,25]; and (3) promoting host genome mutations and DNA damage that may contribute to cellular transformation [26,27,28,29,30,31,32,33,34].

A3-mediated mutagenesis of cellular genomes is hypothesized to contribute to genetic aberrations that lead to cancer [30,41]. This A3 mutator hypothesis is supported by several lines of evidence: (1) A3A and A3B mutation signatures are distinguishable from those caused by other mutagens [29]; (2) A3A and A3B localize to the nucleus [42,43,44] and induce DNA damage [26,45]; and (3) mutation loads correlate with A3A and A3B mRNA expression levels [30,34]. It has been proposed that A3B might be responsible for up to half of all the mutations in breast cancer [46]. A3 mutation signatures are also prevalent in many other different types of cancers, including HPV-associated cervical (CxCa) and head/neck (HNC) cancers [30,31,41,46,47]. Studies from our group and others have shown that A3A and A3B are the only two A3s transcriptionally upregulated in HPV-positive keratinocytes and cancer cells [18,48]. In the context of HPV positive cells, A3A and A3B upregulation is mainly driven by the HPV oncoproteins E7 and E6, respectively. These findings suggest that high levels of E6 and E7 expression during HPV persistence may be a major trigger for A3A- and A3B-mediated mutations. A3A and A3B appear to be primary players in HPV infection and associated cancer mutagenesis, and therefore will be the main focus of this review article.

## 2. Biology of APOBEC3

### 2.1. Structural Features of APOBEC3s

There are seven human A3 genes (*A3A*, *A3B*, *A3C*, *A3D*, *A3F*, *A3G*, and *A3H*) that are arrayed in tandem on chromosome 22 (Figure 1B) [49]. Each of the seven A3 proteins has one (A3A, A3C, A3H) or two (A3B, A3D, A3F, A3G) cytidine deaminase (CD) domains that are characterized by a conserved zinc-coordinating motif (H-X-E-X_23-28_-P-C-X_2-4_-C) (Figure 1C) [50]. Previous research suggested that the C-terminal CD domain (CD2) of the double-domain A3s is catalytically active, while the N-terminal pseudocatalytic domain (CD1), lacking enzymatic activity, is involved in nucleic acid binding (reviewed in References [51,52]). This generalization is largely based on studies of A3F and A3G, and their roles in the inhibition of HIV-1 infection, and may not hold true for other A3s [53,54,55,56]. Bogerd et al. showed that point mutations in either domain significantly limited the ability of A3B to restrict HIV-1 infectivity. This suggests that both CD1 and CD2 of A3B are catalytically active [9]. Interestingly, C-to-T editing of HIV-1 reverse transcripts is still detected if one CD domain of A3B is left functional, but not when both are rendered inactive [9]. However, the ability of both CD1 and CD2 to edit DNA is likely context specific. For instance, mutations in CD1 of A3B had no effect on the overall ability to induce hypermutation of HBV and bacterial DNA, which is in contrast to effects on HIV-1 [9,57]. While both CD domains of A3B may be catalytically active, it is likely that cytidine deamination is either preferentially mediated by CD2 or context specific, in terms of the nature of the substrate recognized.

In contrast to A3B, A3A has a single CD domain that mediates both nucleic acid binding and cytidine deamination. The mechanism by which A3A coordinates both DNA binding and cytidine deaminase activities has recently been uncovered. A3A exists as both a monomer and dimer when in solution and bound to a substrate [58,59]. However, the formation of the homodimer is necessary for high affinity DNA binding [60]. Examination of the A3A crystal structure identified a positively charged groove that is formed upon A3A dimerization. The positively charged amino acids within this groove are positioned to bind to the negatively charged phosphate residues of single stranded DNA (ssDNA) [35,61,62]. These structural studies suggest that the catalytically active form of A3A exists as a homodimer when bound to a substrate.

The substrate specificity of single-domain A3s, like A3A, is likely dependent on relative protein abundance. Bohn et al. hypothesized that A3A, at low protein concentrations, is mostly found in monomeric form and has poor binding affinity for ssDNA. When protein levels are elevated, however, A3A molecules dimerize and deaminate target cytidines with high binding affinity and specificity to ssDNA [60]. On the other hand, the double-domain A3s may have evolved to separate DNA binding from cytidine deamination, resulting in proteins that are more refined to their target. Given that off-target activity of A3s is associated with cancer risk (discussed further in Section 4), targeting the activity of these proteins may be crucial for developing next-generation cancer therapies. Such developments will only be achieved by a thorough understanding of the physiological properties of A3s, which will be greatly aided by mechanistic insights from studies of A3 structure and function.

### 2.2. Evolution of APOBEC3s

**Figure 2:**
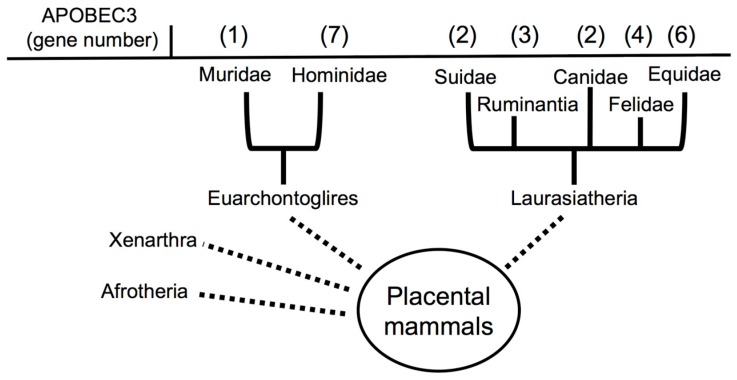
Copy number variation in mammalian *APOBEC3s*. Placental mammals are taxonomically split into four diverse groups (superorders), which include *Afrotheria*, *Xenarthra*, *Euarchontoglires*, and *Larasiatheria*. *APOBEC3s* have been described in the groups belonging to *Euarchontoglires* and *Laurasiatheria*. During mammalian speciation, *A3s* have evolved through a series of gene duplications, fusions, and losses. This has resulted in the copy number variability of mammalian *A3s* during speciation.

The *A3* locus is specific to mammals, yet there is significant variation in the number and arrangement of individual *A3* family members across species (Figure 2) [50]. For instance, primates and rodents are relatives within the superorder of placental mammals, *Euarchontoglires* [63,64,65]. However, primate and rodent genomes contain dramatically different numbers of *A3* genes: seven *A3* genes are encoded in primates while only one is encoded in rodents [49,66,67,68,69]. Additionally, other mammals grouped together within the superorder *Laurasiatheria* [68] have varying numbers of *A3*s, including dog/pig (two) [70], sheep/cow (three) [71], cat (four) [70], and horse (six) [72]. These studies highlight the complex evolutionary trajectory of *A3s* during mammalian speciation, which arose following a series of gene duplications, fusions, and losses (Figure 2). In addition to having an expanded repertoire of *A3s*, primate A3 proteins also display signatures of rapid protein evolution. The rate of non-synonymous amino acid substitutions is significantly greater than synonymous substitutions for several primate A3s [73,74,75,76]. This feature, termed positive natural selection, indicates the existence of a strong selective pressure on the host protein to change and adapt (reviewed in Reference [77]). The relatively rapid expansion of the number of *A3* genes in primates may reflect the necessity, and likely non-redundant function, for specialization of the A3 family members against particular pathogens. Given that A3s restrict viral infections, and that mammalian viruses have evolved countermeasures to avoid A3 restriction, it is plausible that the diversification of primate A3s is a direct response to cope with a wide array of viruses.

### 2.3. Target Specificity of APOBEC3s

While all A3s deaminate cytidine residues, specific dinucleotide motifs are preferably targeted by each A3. The dinucleotide motifs for A3 specificity are immediately 5′ to the target cytidine. For instance, 5′-CC dinucleotides (underline denotes the cytidine targeted for deamination) are the preferred target for A3G [1,78]. In contrast, A3A and A3B preferentially target 5′-TC dinucleotides [61,62,79,80]. By analyzing additional bases adjacent to target cytidine dinucleotides, a recent study further revealed tetra-nucleotide motifs, YTCA and RTCA, differentially targeted by A3A and A3B, respectively [29]. Target site specificity and relative expression levels in different cell and tissue types have been used as a proxy for determining the respective roles of individual A3s on cytidine deamination of both viral and host DNA [20,29,30,81,82].

All seven human A3s bind to ssDNA. A3G targets the minus strand DNA of HIV-1 that is generated during reverse transcription [1,2]. Additionally, A3A potently inhibits the infection of adeno-associated virus (AAV), whose genome consists of a linear ssDNA molecule [11]. However, given that these instances of ssDNA in the cell are relatively rare, additional mechanisms must be available for A3s to target DNA. In theory, the double-stranded genomes of most DNA viruses and their host should be protected from A3-mediated cytidine deamination. Nevertheless, A3-induced mutations have been frequently found in double-stranded DNA (dsDNA) genomes [20,36,82,83]. It has been speculated that transient ssDNA intermediates during gene transcription and genome replication may serve as substrates for A3 deamination. Using the yeast *Saccharomyces cerevisiae* as a model, Hoopes et al. recently demonstrated that A3A- and A3B-mediated mutations are mainly caused by the deamination of the lagging strand template during DNA replication [84]. Consistently, an independent group has shown that A3-associated mutations, identified in sequencing data from The Cancer Genome Atlas (TCGA), are highly enriched on the lagging strand during DNA replication [85]. In addition to ssDNA generated during replication and transcription, replication stress that leads to dsDNA breaks also generates ssDNA substrates that A3A and A3B may act upon [32,42]. These findings imply that cancer cells, given their high levels of cellular proliferation and replication stress, are prime targets for A3A- and A3B-mediated cytidine deamination. This topic is further explored in the sections that follow.

### 2.4. RNA Editing by APOBEC3A

Although ssDNA is the well-known substrate for A3s, recent studies have suggested that A3A can also mutate cellular RNAs [23,24,25]. By analyzing RNA sequencing data from monocytes and macrophages, Sharma et al. discovered widespread C-to-U editing of host cellular mRNAs under hypoxic conditions or after interferon (IFN) treatment [23]. Knockdown of A3A expression reduced RNA editing of succinate dehydrogenase B (SDHB), which was previously shown to be mutated under hypoxic conditions in monocytes [23,86]. Furthermore, editing of cellular RNAs was recapitulated in 293T cells by transient overexpression of A3A [23,24]. The discovery that A3A deaminates RNA, in addition to ssDNA, markedly expands the cellular roles of the A3 family.

Despite numerous antiviral roles for A3A, the precise mechanisms of A3A-mediated restriction are unknown. For instance, transgenic mice expressing human A3A are capable of restricting several murine retroviruses such as mouse mammary tumor virus and murine leukemia virus, yet very minimal DNA deamination was observed [87]. In the context of HPV infection, overexpression of A3A during HPV virion production markedly reduced infectivity [18]. Unexpectedly, despite their restriction being dependent on a functional A3A catalytic domain, no A3A-induced mutations were found in the HPV16 long control region or *E2* gene [18], which were previously identified as A3A mutation hotspots [20,36]. While it is still possible that other regions of the HPV genome may be edited by A3A, RNA editing may provide an alternative mechanism by which A3A restricts HPV infection in lieu of DNA editing. For example, deamination of the transcripts encoding L1 and L2 capsid proteins would likely have a dramatic effect on HPV virion infectivity. Supporting this idea, previous studies have revealed that virus restriction by A3A can occur in a deaminase-dependent mechanism without DNA sequence editing, or by a deaminase-independent mechanism [11,12]. A study from the Malim group further supports this concept by showing that cytidine deamination and DNA editing is not sufficient for antiviral activity during HIV-1 infection [88]. These results suggest alternative mechanisms by which A3A restricts virus infections beyond editing viral DNA sequences. Editing of viral transcripts may provide a novel mechanism by which A3A inhibits virus infection through cytidine deamination.

### 2.5. Transcriptional Regulation of APOBEC3A and APOBEC3B

The innate antiviral immune response is commonly initiated by cellular sensors that recognize foreign entities. These sensors relay intracellular signals to their effectors, which are responsible for clearing invading pathogens from the host cell. The A3 family members are important effectors of the innate antiviral immune defense (reviewed in Reference [89]). The detection of viral factors by cellular sensors leads to the activation of type 1 IFN signaling [90], which upregulates the expression of numerous antiviral genes, including the *A3* family members [36,38,91,92,93]. A3B transcription also appears to be activated through protein kinase C and nuclear factor kappa-light-chain-enhancer of B cells (NF-κB) signaling [94,95]. When expressed at high levels, A3s are capable of limiting infection of a diverse range of viruses, including retroviruses and DNA viruses. We and others have demonstrated that A3A and A3B transcription is also upregulated by the HPV oncoproteins E6 and E7 [18,48]. Interestingly, analyses of gene expression data from patient tissue specimens and cell lines confirm that A3A and A3B mRNA levels are elevated in HPV-positive cancers compared to normal tissues [18,30,48,96]. Together, these studies suggest that HPV infection induces the expression of A3A and A3B.

The *A3B* promoter is composed of a distal region for basal transactivation and a proximal region for transcriptional repression [97]. Interestingly, both the distal and proximal regions contain E6-responsive elements, which are essential for *A3B* promoter activity. DNA pull-down and chromatin immunoprecipitation assays further identified zinc finger protein 384 (ZNF384) as an important player for HPV-induced *A3B* transactivation [97]. Additionally, the same group has reported that HPV16 E6 upregulates *A3B* transcription by enhancing the expression of transcriptional enhancer factor (TEA) domain (TEAD) transcription factor that then binds to the *A3B* promoter [98]. These results suggest that *A3B* transcription may be directly activated by HPV16 E6. However, it is still possible that increased A3A and A3B mRNA levels may be, at least in part, due to an inadvertent consequence of a master transcription factor dysregulated by the HPV oncoproteins.

It has been speculated that HPV16 E6 expression increases *A3B* transcription through the functional inactivation of p53 [48]. Consistent with this notion, it was recently shown that wildtype p53 represses *A3B* transcription, and inactivating mutations in p53 protein leads to the upregulation of A3B expression [30,99,100]. In contrast to A3B, a study showed that A3A expression is upregulated by activated p53 [99]. A3A alters genome integrity by inducing DNA strand breaks and activating the DNA damage response [26,42], resulting in cell cycle arrest and apoptosis closely linked to p53 responses. As it is possible that the p53 regulation of A3 expression influences the course of a viral infection under conditions of cellular stress, understanding the interactions between p53 and A3s may provide novel avenues to treat persistent viral infections and neoplastic lesions.

## 3. Restriction of DNA Viruses by APOBEC3A

### 3.1. APOBEC3A Restriction of HPV Infection

A3A is localized throughout the cell in both cytoplasmic and nuclear compartments [26,42,43]. A growing body of evidence has implicated A3A as an important contributor to somatic mutations in human genomes [29], further emphasizing that A3A has access to nuclear DNA. Access to the nucleus may partially confer specificity to the types of viruses targeted by A3A (discussed further below). A3A is expressed in several cell types, including keratinocytes and myeloid cells [10,20,36,101,102]. Particularly, we have found that compared to cutaneous skin, A3A is expressed at high levels in mucosal tissue, which is vulnerable to the entry of foreign invaders like viruses [103].

**Figure 3:**
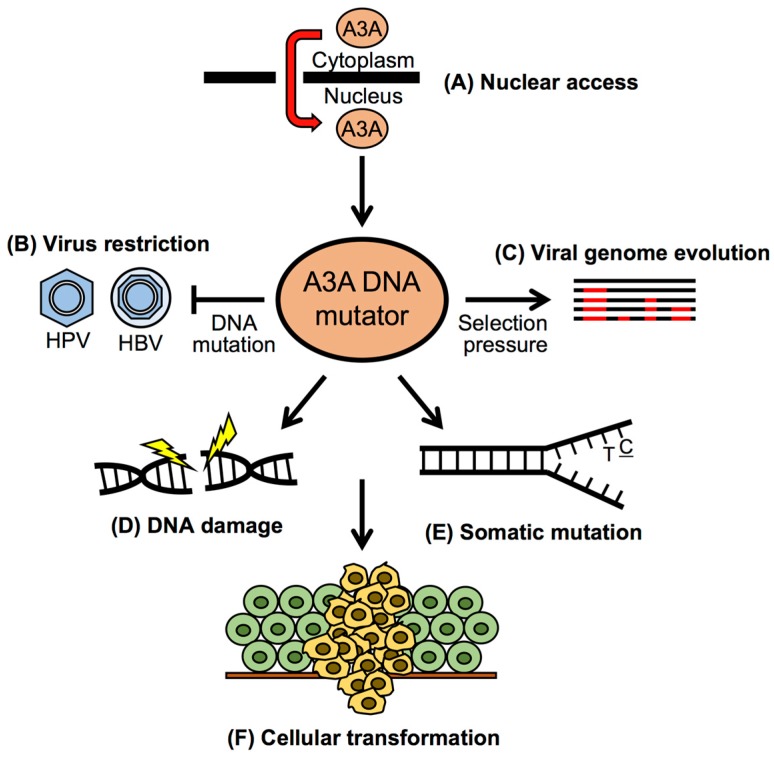
A3A is arguably the most important A3 family member in HPV restriction, evolution, and cancer mutagenesis. (**A**) A3A localizes to both the cytoplasm and nuclear compartments. Nuclear access broadens the substrates targeting by A3A; (**B**) In addition to restricting lentiviruses during reverse transcription in the cytoplasm, A3A also restricts DNA viruses that replicate in the nucleus, such as human papillomavirus (HPV) and hepatitis B virus (HBV); (**C**) Selection pressures imposed by A3A may lead to viral genome evolution (pictured in red) and partial escape from A3A restriction, as has been proposed for HPV [103]; (**D**,**E**) A3A activity enhanced by virus persistence and/or chronic inflammation may promote DNA damage and induce somatic mutations in host DNA; (**F**) Somatic mutagenesis and DNA damage further enables cancer cell evolution and drives disease progression.

Recent findings suggest that A3A is arguably the most important A3 family member targeting foreign DNA (Figure 3). A3A can localize to the nucleus [26,42,43], binds to ssDNA with high affinity [59,61], and deaminates cytidines in transient ssDNA undergoing transcription or replication [84,104]. The partially single-stranded genomes of HBV and AAV are highly susceptible to A3A restriction [11,17,37,81,105]. Several lines of evidence suggest that HPV genomes are also targeted by A3A [20,96,103,106,107]. Based on substrate specificity (TC dinucleotide targets) and high A3A expression levels in keratinocytes (the host cell for HPV infection), Vartanian et al. provided the first in vitro and in vivo evidence to suggest that A3A is a mutator of HPV genomes [20]. Since this seminal study, there have been multiple reports of HPV genome editing in patient tissue biopsies, including CxCa and oropharyngeal cancers [96,106,107]. These studies highlight that A3A may play an important role in restricting HPV infection.

Interferon-β (IFN-β) treatment significantly restricts HPV infection in keratinocytes as well as represses HPV DNA replication in infected keratinocytes [18,108,109,110,111]. Given that A3s are IFN-inducible proteins that target retroviruses and DNA viruses, Wang et al. sought to clarify whether A3s are also involved in the IFN-β-mediated response against HPV infections. This study revealed that IFN-β treatment upregulated A3A expression in cervical keratinocytes, and that knockdown of A3A expression reduced IFN-β-induced hypermutation of the viral *E2* gene [36]. However, A3A-induced hypermutation was detected only after enrichment by differential DNA denaturation PCR (3D-PCR), indicating that A3A-induced mutation events are rare. These interesting discoveries led us to question whether A3A affects HPV infectivity. Using our high-yield HPV production system [112], we have shown that virions packaged in cells overexpressing A3A is dramatically less infectious in keratinocytes. In contrast, the expression of other nuclear-localized A3s, A3B and A3C, had no effect on restricting viral infection [18]. HPV restriction by A3A is deaminase dependent, as a catalytically inactive mutant A3A was unable to restrict HPV infection [18]. However, using highly sensitive next-generation sequencing, we were unable to detect A3A-induced mutations in genomic regions previously shown to be edited by A3s [18,20,36]. Further analysis of whole viral genome or RNA sequences may identify critical A3A mutation targets that disrupt HPV infectivity.

### 3.2. APOBEC3A-Mediated Clearance of HPV DNA during Persistent Infection

A3A also plays an important role in mediating the clearance of foreign, circular DNA from cells [21,22]. Stenglein et al. have shown that A3A overexpression leads to the deamination and degradation of transfected foreign plasmid DNA [22]. Both HBV and HPV genomes are maintained as dsDNA episomes in the nucleus of persistently infected cells. Given that A3A is nuclear and restricts foreign circular DNA, it is possible that A3A may mediate the clearance of HBV and HPV DNA in persistently infected cells. Indeed, Lucifora et al. have shown that IFN-α-induced A3A triggers cytidine deamination and degradation of nuclear HBV DNA [16]. Confocal microscopy revealed that A3A colocalizes with the HBV core protein, which likely facilitates close contact with viral DNA in the nucleus. The degradation of HBV DNA prevents virus reactivation without hepatotoxicity [16], suggesting that A3A may be used as a tool for the treatment of persistent HBV infection, for which current therapies are limited. Accordingly, it would be of immense interest to the HPV field to determine whether A3A can similarly clear persistent HPV infection. In a series of elegant studies, the Coleman group has identified important roles for antiviral IFN responses in clearing persistent HPV infection, showing that IFN-β treatment of HPV-positive CxCa cells leads to a rapid loss of viral episomes [108,113]. In addition, loss of HPV DNA in CxCa cells during serial passaging is correlated with a surge in expression of IFN-inducible antiviral genes. This suggests that the antiviral IFN response is likely one of the key contributors in promoting spontaneous loss of extrachromosomal HPV DNA [113]. Along with other similar results, these findings collectively suggest that antiviral IFN responses likely play an important role in limiting the persistence of extrachromosomal HPV DNA [114,115,116]. Given that A3A is an IFN-inducible protein in keratinocytes [36], and that A3A can eliminate foreign DNA [22], it is plausible that A3A may contribute to the loss of HPV genomes in persistently infected cells, similar to clearance of HBV DNA [16]. However, the restriction pressure of IFN and A3A may also accelerate HPV-induced cancer progression by facilitating the integration of HPV DNA into the host chromosome. Kondo et al. have found that A3A expression is strongly linked to HPV integration in oropharyngeal cancers [96]. Similarly, either IFN-β or IFN-γ treatment significantly enhances HPV integration in persistently infected cervical keratinocytes [108,115]. These findings suggest that using IFN and/or A3A may not be feasible as antiviral agents to treat patients. Developing therapeutics to treat chronic HPV infections requires an in-depth understanding of the complicated interactions between A3A and HPV during persistent infection.

### 3.3. Viral Evasion of APOBEC3A-Mediated Restriction

Given the antiviral potency of A3A, it is likely that viruses have evolved countermeasures to combat or avoid A3A-mediated restriction. For instance, A3A significantly inhibits HIV-1 replication following infection of myeloid cells [10]. Interestingly, the viral accessory proteins Viral infectivity factor (Vif) and Viral protein X (Vpx) of HIV-1 and simian immunodeficiency virus of macaques (SIVmac), respectively, are capable of degrading A3A protein [10]. A3A degradation by viral proteins appears to be conserved in primate lentiviruses, further emphasizing the importance of antagonizing A3A during lentiviral infection. Similarly, as A3A restricts HPV infectivity, elevated A3A expression in HPV-infected cells is likely to be deleterious for viral fitness. Thus, one would predict that HPV has evolved strategies to evade A3A-mediated restriction. It is well known that the HIV-1 accessory protein Vif degrades another A3 family member, A3G, through a ubiquitin mediated, proteasome-dependent process that requires the cellular factors cullin 5, elongin B/C, and Ring-box protein 1 (RBX1) [117,118,119]. Interestingly, high-risk HPV E7 coordinates a similar process whereby interactions with the cullin 2 ubiquitin ligase complex, which also contains elongin B/C and RBX1, mediates the degradation of the tumor suppressor retinoblastoma protein (pRB) [120,121,122]. Given these striking similarities, it stands to reason that high-risk HPV E7 might facilitate the degradation of A3A protein in a process similar to HIV-1 Vif. Contrary to this hypothesis, we found that the expression of high-risk HPV E7 in keratinocytes significantly increased A3A protein levels [123]. While this relative increase in A3A protein may well explain the A3 mutation signatures in HPV-positive cancers, it implies that HPV likely employs other mechanisms to cope with elevated A3A levels during persistent infection.

As explained above, A3s deaminate cytidines within the context of preferred dinucleotides. A3A, for instance, prefers targeting cytidine residues that are preceded by thymidine (5′-TC). An alternative mechanism to evade A3A deaminase activity would be to reduce the prevalence of A3A target sequences within the viral genome. Underrepresentation of CG dinucleotides in small DNA viruses, including polyomaviruses and papillomaviruses, has been suggested as a means to evade toll-like receptor 9 (TLR9) recognition and/or host DNA methylation of viral genomes [103,124,125,126,127]. As papillomaviruses have co-evolved with their host over millions of years, it is possible that A3A has exerted selective pressure on papillomavirus evolution, resulting in reduced TC dinucleotide contents in viral genomes (Figure 3). Analysis of 274 papillomavirus genomes has revealed that CG and TC dinucleotides are significantly depleted in all papillomavirus genomes [103]. Interestingly, the magnitude of TC depletion is greater in HPV genotypes from the *Alphapapillomavirus* genus (α-HPV) than β- or γ-HPV genotypes, while the degree of CG depletion is similar across all HPV genera. The significant difference in TC depletion between α- and β/γ-HPVs may be caused by either (1) the ancestral *Alphapapillomavirus* having low TC contents that subsequently radiated to all extant genotypes, or (2) a strong selective pressure that was exerted on the entire *Alphapapillomavirus* clade leading to extreme TC depletion. Phylogenetic reconstruction of the ancestral *Alphapapillomavirus* state revealed that the most recent common ancestor of all *Alphapapillomavirus* had TC contents significantly higher than extant members of this clade [103]. Thus, TC depletion likely occurred after *Alphapapillomavirus* began to diverge. This finding is suggestive of a possible role for A3 restriction that drove TC depletion within this clade. One major hallmark of most α-HPVs is tropism for mucosal tissues, while β- and γ-HPVs are typically found in association with cutaneous skin [128]. Analysis of publicly available RNA sequencing data revealed that A3A expression levels are significantly higher in mucosal skin compared to cutaneous skin [103]. Taken together, these findings suggest an evolutionary model in which HPV copes with elevated A3A expression by limiting the number of TC dinucleotides within their genomes [129]. In addition to the reduction of A3A target motifs, HPV may employ other means of escaping restriction by A3A. Further studies may provide additional clues about the complex interactions between HPV and A3s.

## 4. Cancer Mutagenesis by APOBEC3A

### 4.1. Sources of APOBEC3 Mutational Signatures in HPV-Positive Cancer

Although many studies have identified A3 mutational signatures in multiple human cancers, the molecular triggers resulting in off-target A3 activity on cellular DNA remain poorly understood. We and others have reported that the mRNA levels of A3A and A3B are significantly higher in HPV-positive keratinocytes and cancer tissues compared to uninfected keratinocytes and normal tissues, respectively [18,48]. A3A mRNA expression is increased by high-risk HPV E7, while A3B mRNA expression is upregulated by both the HPV oncoproteins E6 and E7 in cultured keratinocytes and human tonsillar epithelial cells. Interestingly, our results have shown that the mRNA expression levels of A3A and A3B are highly correlated, indicating that A3A and A3B may share common mechanisms for transcriptional regulation [18]. While mRNA expression of A3A and A3B has been well studied, little is known about their protein levels and posttranslational modification. Our unpublished study found that A3A protein levels are dramatically increased in human keratinocytes by high-risk HPV E7 mediated protein stabilization [123]. Consistent with these results, analysis of exome sequencing data from TCGA has revealed that HPV-positive HNC genomes contain high levels of A3-mediated driver mutations, while HPV-negative HNC displays a smoking-associated mutational signature [130]. Further, A3 deaminase activity is causally associated with helical domain hotspot mutations in the phosphatidylinositol-4,5-bisphosphate 3-kinase catalytic subunit alpha (*PIK3CA*) gene, which are more prevalent in HPV-positive cancers when compared to HPV-negative cancers [131]. Taken together, these findings strongly suggest that HPV oncoprotein expression during persistent viral infection may be the trigger for the increase of A3A and A3B expression, culminating in the accumulation of somatic mutations in HPV-positive cancers (Figure 3).

### 4.2. The Relative Contributions of APOBEC3A and APOBEC3B to Cancer Mutagenesis

The expression of the HPV oncoproteins E6 and E7 immediately inactivates numerous cellular proteins, including the tumor suppressors p53 and pRB [132,133]. While these mechanisms are important for promoting cell proliferation, they alone are not sufficient to drive cancer progression. The accumulation of additional somatic mutations over decades of persistent infection is necessary for cancer progression. Recent findings suggest that A3A and/or A3B potentially play important roles in cancer mutagenesis by mutating host genomic DNA [30,31,41,46,47,134]. Since both A3A and A3B are upregulated in HPV-positive epithelial cells and target the same TC dinucleotide motif, it is difficult to tease out the relative contributions of either in promoting somatic mutations in cancer cell genomes. Previous studies have correlated elevated A3B mRNA expression with A3-associated mutation loads, and proposed A3B as the source of A3 mutation signatures in cervical, bladder, lung, head and neck, ovarian, and breast cancers [30,47,135,136]. Burns et al. further showed that knockdown of A3B expression by short-hairpin RNA (shRNA) abrogates cytidine deaminase activity in the lysate of a breast cancer cell line [41]. Contrary to these findings, recent studies have shown that the *A3B* deletion polymorphism, highly prevalent in South East Asia, China, and Oceania, is associated with increased risk of breast and ovarian cancer [136,137,138]. Caval et al. revealed that *A3B* deletion frequently generates a chimeric *A3A–A3B* deletion allele in which the *A3A* gene is fused to the 3' untranslated region (UTR) of *A3B* [139]. The mRNA generated from the chimeric *A3A–A3B* deletion allele is more stable than wildtype A3A transcripts, and the resulting protein facilitates DNA damage. Using yeast models, Chan et al. recently found that A3A and A3B mutation signatures may be distinguishable by the preferred target motifs: YTCA favored by A3A and RTCA favored by A3B [29]. Further analysis of sequence data from yeast and human cancer genomes uncovered that A3A-like mutations are 10 times more abundant than A3B-like mutations. Based on these results, the authors propose that mutagenesis and DNA damage caused by A3A might be greater than A3B in HPV-associated cancers. Interestingly, A3A expression is tightly correlated with HPV DNA integration into host chromosomes, which is facilitated by dsDNA breaks [96,140]. Consistently, A3A protein dramatically accumulates in HPV-positive keratinocytes by E7-mediated protein stabilization [123]. Taken together, these findings suggest that both A3A and A3B may contribute to cancer mutagenesis, but likely have differential contributions in various cancers when triggered by different mechanisms.

### 4.3. Source of APOBEC3 Signature in Other Virus-Associated Cancers

Type I IFNs, commonly induced during various virus infections, highly upregulate A3A and A3B expression. This suggests that persistent inflammatory responses may generally facilitate somatic mutations by A3A and A3B [16,18,38,92]. A3B is also upregulated by several polyomaviruses (PyV), including JC PyV, Merkel cell PyV, and BK PyV through a mechanism dependent on large T antigen expression [141]. These results suggest that A3 mutation signatures from A3A and A3B may also be caused by other virus infections as well. However, it is not clear whether the increase of A3B expression observed in many HPV-negative cancers is mediated by type I IFN or by other factors such as viral proteins similar to the HPV oncoproteins. Further investigations are required to determine the mechanisms by which A3A and A3B are activated and contribute to cancer mutagenesis.

### 4.4. APOBEC3-Mediated Somatic Mutations and Clinical Outcomes of HPV-Positive Cancers

**Figure 4:**
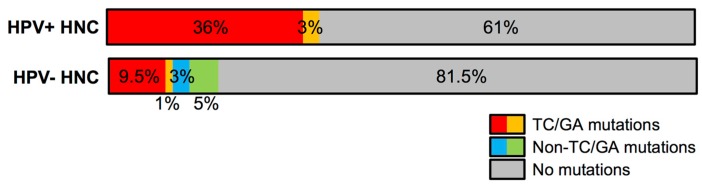
High levels of TC/GA mutations in the phosphatidylinositol-4,5-bisphosphate 3-kinase catalytic subunit alpha (*PIK3CA*) open reading frame in HPV-positive head and neck cancers (HNCs) compared to HPV-negative HNCs. Amino acid mutation data of PIK3CA in HNC patients was obtained from The Cancer Genome Atlas through cBioPortal (cbioportal.org) [149]: HPV-positive HNC (HPV+ HNC), *n* = 36; HPV-negative HNC (HPV- HNC), *n* = 243. Amino acid changes by TC/GA mutations are indicated as red (E542K and E545K) and orange (M1043V, R88Q, G1007R, G451R, R335G), and amino acid changes by non-TC/GA mutations are indicated as blue (H1047L, H1047R) and green (C604R, C901F, C971R, E110del, E365V, G363A, K111E, K111N, Q75E, R975S, V344G, W328S). Shown are the percentage of patients containing each mutation signature.

Somatic mutagenesis has been recognized as a key mechanism of carcinogenesis by generating driver mutations in numerous genes including *p53*, epidermal growth factor receptor (*EGFR*), *pR*B, and *PIK3CA* [142,143]. Given that the deaminase activity of A3A and A3B in epithelial cells mutates the TC motifs of host DNA as well as viral DNA, somatic mutagenesis by A3A and A3B is likely associated with cancer risk. Indeed, a high frequency of activating *PIK3CA* mutations was observed in HPV-positive HNCs compared to HPV-negative HNCs [131,144]. Our analysis also showed that all *PIK3CA* mutations in HPV-positive HNC, dominant with the E542K and E545K substitutions, are caused by GA-to-AA changes, while only about a half of *PIK3CA* mutations in HPV-negative HNC are from GA-to-AA changes (Figure 4). As *PIK3CA* is an oncogenic driver gene, A3A- and/or A3B-mediated somatic mutations may contribute to HPV-associated cancer progression through mutations in *PIK3CA*.

Contrary to the idea that A3-mediated somatic mutations may drive HPV-positive cancer progression, recent cancer immunology studies have shown that high levels of somatic mutations favor antitumor immune responses that also coincide with better prognosis after immunotherapies [145,146,147]. In these instances, tumor neoantigens are recognized as emerging targets for personalized cancer immunotherapies. This implies that cancers with a high degree of A3 mutation signatures may be beneficial for immunotherapies that induce robust antitumor T cell responses specific to neoantigens generated by A3-mediated mutations. A recent study has revealed that tumor infiltrating lymphocytes in CxCa are more reactive to neoantigens than to HPV viral epitopes [148]. This suggests that abundant neoantigens in HPV-positive cancers may be associated with the deaminase activity of upregulated A3A and A3B expression. If this is true, A3-mediated mutations could be utilized beneficially to identify T cell epitopes and treat HPV-positive cancer patients. Thus, it would be interesting to investigate if A3 mutation loads in patients correlate to better outcome following current immunotherapies with immune checkpoint blockades.

## 5. Conclusions and Perspectives

The inactivation of tumor suppressors by HPV oncoproteins is robust and quick. For example, p53 and pRB are degraded in host cells in which high-risk HPV E6 and E7 are expressed [132,133]. Nevertheless, HPV-associated cancer progression is a slow process, typically taking two to three decades. A growing number of studies have shown that the continuous expression of E6 and E7 is required through the full process of cancer progression and maintenance [150,151,152,153,154,155,156]. Our CxCa progression study has shown that many HPV-specific gene expression changes occur in a later stage or continuously throughout decades of cancer progression [157]. In this regard, the roles of A3A and A3B in HPV-associated cancer progression are particularly interesting. However, most of these new findings have generated more questions than answers, particularly due to the causal relations and the need for defining the mechanistic elements of these interactions. Now, most studies on A3-induced cancer mutagenesis have been limited to using highly biased sequencing approaches or are based on correlations between expression levels and preferred target sequence changes. Since A3-induced somatic mutations probably accumulate over decades, it would be technically challenging to recapitulate and confirm this process in experimental models. To overcome these barriers, developing transgenic animal models expressing human A3s along with HPV oncoproteins may provide useful tools to track cancer mutagenesis. Additionally, further work is needed to elucidate the mechanisms of A3A restriction of HPV infection, which are distinct from A3B and A3C. It would also be interesting to further investigate whether A3A and A3B restrict other small DNA tumor viruses and contribute to somatic mutagenesis of their associated cancers. Future studies may provide great insights into how virus-host interactions drive the evolution of viruses and host cells, and how these interactions may lead to unexpected consequences such as cancer development.

## Figures and Tables

**Table 1:** Restriction of DNA viruses by APOBEC3 family members.

Virus	APOBEC3 Family Members	Functions on Viral Genome	References
**Parvovirus**	A3A	Unknown	[11,12,13,35]
**Herpesvirus**	A3A, A3G	DNA editing, unknown	[14,15]
**Papillomavirus**	A3A, A3C (?)	DNA editing, unknown	[18,20,36]
**Hepadnavirus**	A3A, A3B, A3C, A3F, A3G, A3H	DNA editing, deamination, and degradation	[16,17,37,38,39,40]
